# Mendelian Randomization Study Shows No Causal Relationship Between Circulating Urate Levels and Parkinson’s Disease

**DOI:** 10.1002/ana.25294

**Published:** 2018-08

**Authors:** Demis A. Kia, Alastair J. Noyce, Jon White, Doug Speed, Aude Nicolas, IPDGC collaborators, Stephen Burgess, Debbie A. Lawlor, George Davey Smith, Andrew Singleton, Mike A. Nalls, Reecha Sofat, Nicholas W. Wood

**Affiliations:** 1 Department of Molecular Neuroscience, UCL Institute of Neurology, London, United Kingdom; 2 Preventive Neurology Unit, Wolfson Institute of Preventive Medicine, Queen Mary University of London, London, United Kingdom; 3 UCL Genetics Institute, University College, London, United Kingdom; 4 Laboratory for Neurogenetics, National Institutes for Health, Bethesda, MD; 5 Department of Public Health and Primary Care, University of Cambridge, Cambridge, United Kingdom; 6 MRC Integrative Epidemiology Unit at the University of Bristol, Bristol, United Kingdom; 7 Population Health Science, Bristol Medical School of Bristol, Bristol, United Kingdom; 8 Data Tecnica International, Glen Echo, MD; 9 Institute of Health Informatics, University College London, London, United Kingdom

## Abstract

**Objective:**

Observational studies have shown that increased plasma urate is associated with lower risk of Parkinson’s disease (PD), but these studies were not designed to test causality. If a causal relationship exists, then modulating plasma urate levels could be a potential preventive avenue for PD. We used a large two-sample Mendelian randomization (MR) design to assess for a causal relationship between plasma urate and PD risk.

**Methods:**

We used a genetic instrument consisting of 31 independent loci for plasma urate on a case-control genome-wide association study data set, which included 13,708 PD cases and 95,282 controls. Individual effect estimates for each SNP were combined using the inverse-variance weighted (IVW) method. Two additional methods, MR-Egger and a penalized weighted median (PWM)-based approach, were used to assess potential bias attributed to pleiotropy or invalid instruments.

**Results:**

We found no evidence for a causal relationship between urate and PD, with an effect estimate from the IVW method of odds ratio (OR) 1.03 (95% confidence interval [CI], 0.88–1.20) per 1-standard-deviation increase in plasma urate levels. MR Egger and PWM analyses yielded similar estimates (OR, 0.99 [95% CI, 0.83–1.17] and 0.99 [95% CI, 0.86−1.14], respectively).

**Interpretation:**

We did not find evidence for a linear causal protective effect by urate on PD risk. The associations observed in previous observational studies may be, in part, attributed to confounding or reverse causality. In the context of the present findings, strategies to elevate circulating urate levels may not reduce overall PD risk.

Parkinson’s disease (PD) is the second-most prevalent neurodegenerative disorder,^1^ affecting 1.2 in 100 individuals aged >65 years in the United States.^2^ Observational studies indicate that several modifiable risk factors may be associated with PD, and, if causal, these could offer novel therapeutic targets.^3^,^4^ However, residual confounding, reverse causality, and regression dilution bias can limit causal inferences drawn from observational studies.^5^

Urate is a metabolite of human purine metabolism produced by action of the enzyme, xanthine oxidoreductase, on hypoxanthine and xanthine. High plasma concentrations of circulating urate are associated with gout, the deposition of monosodium urate crystals in joints, soft tissues, and renal parenchyma. The causal role of urate in gout has been previously demonstrated using Mendelian randomization (MR).^6^,^7^

It has been suggested that high circulating urate concentration may protect against PD. The putative mechanism is thought to be associated with the antioxidative properties of urate, which may have a neuroprotective effect by scavenging reactive oxygen and nitrogen species and acting as an iron chelator, rescuing cells from oxidative stress.^8^–^11^ Observational studies and meta-analyses have tended to show a negative association between urate and PD incidence, with some suggesting that this effect is stronger in men compared to women, though these studies were mostly small in size and suffered from high heterogeneity.^12^–^14^ No randomized controlled trial data currently exist to confirm a causal relationship between urate and PD. An ongoing clinical trial investigating inosine, a urate precursor, as disease-modifying therapy for PD, is due to report its findings in 2020.^15^,^16^ MR has been explored as a means to investigate the relationship between plasma urate and PD incidence, progression, and age at onset, with some evidence to support a protective role.^17^–^20^ Overall, the size of these studies has also tended to be small and they had limitations inherent to observational study designs, which are minimized in a formal instrumental variable analysis. To fully understand the causal relationship between urate and PD, further study is warranted.

Here, we used MR to determine whether there is evidence for a causal relationship between plasma urate concentration and risk of PD. Where genetic variants are robustly associated with potential risk factors, an MR approach can be used to provide an unbiased and unconfounded effect estimate, which can provide evidence of causality. This is because genotype is not modifiable by disease, thus decreasing the likelihood of bias by reverse causality, and the random allocation of alleles at gametogenesis reduces the likelihood of confounding by socioeconomic and lifestyle characteristics that tend to bias conventional multivariable regression analyses.^21^ Here, we use two-sample MR to test the effect of plasma urate on PD, using a multilocus instrument on a genome-wide association study (GWAS) sample of up to 13,708 PD cases and 95,282 controls.^22^,^23^ To minimize the possibility that results were obscured by pleiotropy in our instrument and ensure that the results are not biased because of violations of the MR assumptions, we performed two additional MR analyses beyond the traditional inverse variance weighted (IVW) method: MR Egger and a penalized weighted median (PWM)-based approach.^24^,^25^

## Subjects/Materials and Methods

We used publicly available genetic summary association data with appropriate institutional review board (IRB) and ethical review. Separate IRB/ethical review was not required for this study.

### Genetic Instrument Development

We used an established genetic instrument for urate, consisting of 31 single-nucleotide polymorphisms (SNPs) that have been associated with plasma urate levels in GWAS meta-analyses in populations with European ancestry.^26^ The construction of this instrument has been described previously.^26^ Briefly, 31 independent loci (R^2^ < 0.3; separated by >140 kb), with an association with urate at *p* < 5 × 10^−8^ or *p* < 5 × 10^−7^ with a clear functional role in urate metabolism, were identified. The lead SNP from each locus was picked as the instrumental variable for that locus, and its published effect size and standard error were noted. For SNPs where data were available from multiple independent publications and cohorts, effect estimates were combined using fixed-effects meta-analysis.^27^–^29^ Effect size represents a standard deviation (SD) increase in plasma urate levels per allele. A subset of 26 loci from our instrument explained 7% of variance in urate concentrations in the Global Urate Genetics Consortium (GUGC) GWAS, with 3.4% explained by the *SLC2A9* and *ABCG2* loci alone.^27^ This multilocus approach allows us to evaluate our instrument for pleiotropy and ensure that the results are not attributed to violations of the MR assumptions. SNPs in the instrument have been summarized in Table 1.

### PD Genetic Data

Summary statistics from the discovery phase of a GWAS meta-analysis of PD were used including 7,893,273 genotyped and imputed variants in 13,708 PD cases and 95,282 controls of European ancestry from 15 studies. Details on recruitment and diagnostic assessment as well as quality-control procedures of the GWAS are described in the original publication.^23^ All 31 SNPs in the instrument were present in the PD genetic data set. For purposes of this study, quality control included ensuring no strand mismatches and alignment of SNP effect sizes with respect to urate increasing allele. A palindromic SNP, rs17632159, in the PD data set was reconciled comparing allele frequencies in the PD and GUGC data sets to ensure that effect estimates were recorded with respect to the same allele (C-allele had a frequency of 0.31 in both data sets).

### Instrumental Variable Analysis

Effect estimates for the association between a genetically related 1-SD increase in plasma urate level and the odds ratio (OR) for PD were obtained for each SNP in the instrument separately using the Wald ratio method.^30^ Individual effect estimates were then combined using the IVW method, constraining the weighted regression line to pass through the origin. This method yields an effect estimate that converges with the estimate obtained from the two-stage least squares method with individual-level data.^31^

A key assumption of any instrumental variable analysis, including MR, is that the instrumental variable(s) (genetic variants in MR) are not associated with the outcome in any other way other than through the exposure under analysis. Violation of this assumption in MR is most commonly attributed to horizontal pleiotropy (one or more genetic instruments affecting other characteristics that are risk factors for the outcome, independent of the main exposure of interest). We used three methods to explore the susceptibility of our effect estimates to such bias. First, we examined the heterogeneity between the effect estimates from individual SNPs, through Cochran’s Q test, which is able to detect moderate to weak pleiotropy.^32^

Second, we repeated the analysis using the MR Egger method (rather than IVW method) to combine individual SNP estimates.^24^ This method is similar to IVW, but does not constrain the regression line of mean urate and mean PD levels for each SNP to go through zero. A nonzero intercept with MR Egger regression implies the presence of net directional pleiotropy which may bias the IVW estimate, and the slope from MR Egger is the effect estimate having relaxed the assumption of bias attributed to pleiotropy. Whereas the assumption of no other path from the genetic instrument to outcome, other than by the risk factor of interest, can be relaxed with MR Egger, this method has an additional assumption. The Instrument Strength Independent of the Direct Effect (InSIDE) assumption of MR Egger will be violated if the genetic instrument/risk factor association (here joint association of the 31 SNPs with urate) is correlated with any pleiotropic associations from the SNPs to the outcome.

Third, we used an alternative method that has different assumptions regarding pleiotropy to both the IVW or MR Egger estimates. The PWM-based method gives consistent effect estimates under the assumption that no more than 50% of the weight of the MR effect estimate, where weight is determined by the magnitude of their association with risk factor (here urate), is from invalid (eg, pleiotropic) SNPs.^25^ Given that these three MR methods have different assumptions and therefore different key sources of bias, where results are consistent across the three this supports that consistent result being the true causal effect.^33^ To assess the possibility that the overall effect estimate was being driven by any particular SNP in the instrument, we iteratively removed each SNP from the instrument and reran the MR analysis to assess whether the effect estimate is robust to removing any individual SNP from the instrument.

To assess the power in our study to precisely detect a causal effect, we used a published method for power calculations for MR studies with a binary outcome.^34^ For this calculation, we assumed a proportion of variance explained by our instrument of 7%, as reported in the GUGC study for the subset of 26 SNPs in our instrument.

### Statistical Analysis

All statistical analysis was performed using R software (version 3.2.4; R Foundation for Statistical Computing, Vienna, Austria).

## Results

**Figure 1:**
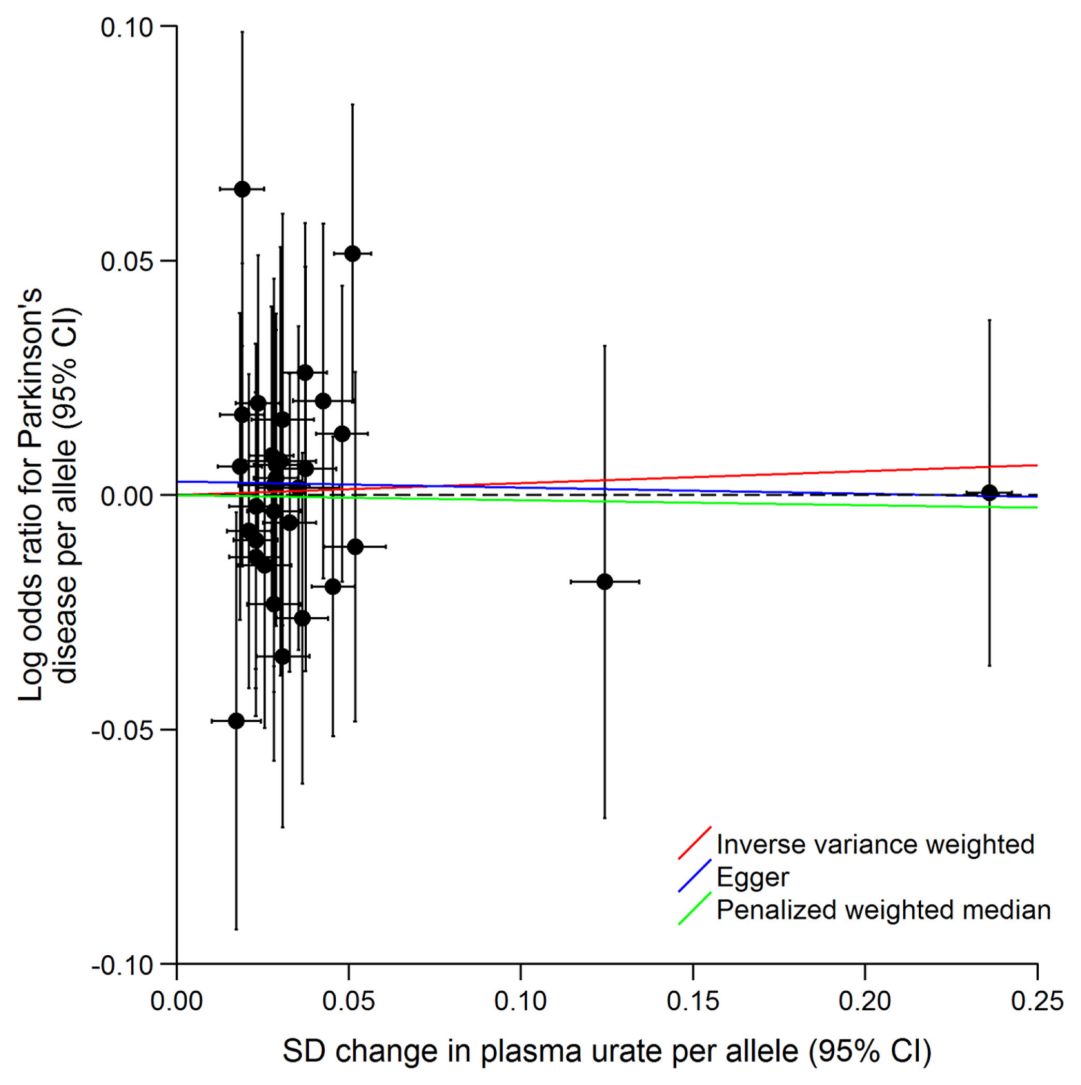
Scatter plot of results from the instrumental variable analysis for individual SNPs and pooled estimates. CI = confidence interval; SD = standard deviation; SNPs = single-nucleotide polymorphisms.

The main IVW MR analysis did not provide clear evidence for a causal effect between plasma urate and PD risk (OR per 1-SD increase in urate concentration was 1.03 [95% confidence interval {CI}, 0.88–1.20]; Fig 1). No individual SNP produced an estimate that would be consistent with a protective effect. Effect estimates from two SNPs (rs1165151 and rs2307394) suggested an increased risk of PD with increased plasma urate concentration. The SNP from the *SLC2A9* locus (rs12498742), which was used as the sole locus in a previous MR study, produced an effect estimate consistent with no effect of urate on PD risk (OR, 1.00 [95% CI, 0.86−1.17]).^17^

**Figure 2:**
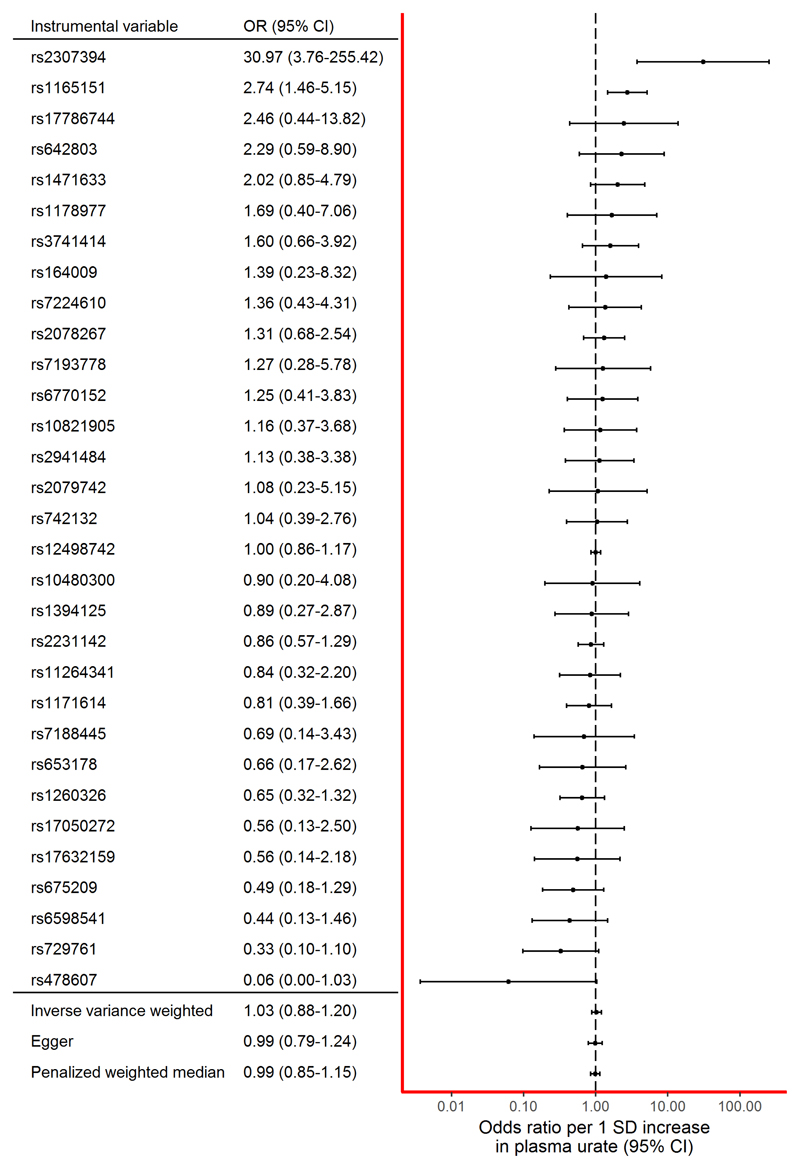
Forest plot of the association of individual SNPs with urate and PD risk, together with pooled estimates. CI = confidence interval; OR = odds ratio; PD = Parkinson’s disease; SD = standard deviation; SNPs = single-nucleotide polymorphisms. [Color figure can be viewed at www.annalsofneurology.org]

There was weak evidence of heterogeneity among the instrumental variable estimates from individual SNPs (Cochran’s Q = 43.06; *p* = 0.07). MR Egger analysis produced a y-intercept of 0.00 (95% CI, –0.01 to 0.02; *p* = 0.63), suggesting that any potential pleiotropy in the instruments was balanced and unlikely to bias the results of the analysis. The causal effect estimate derived from MR Egger was OR 0.99 (95% CI, 0.83–1.17) per 1-SD increase in urate. The PWM analysis yielded an almost identical effect estimate to the one with the traditional IVW method and MR Egger (OR, 0.99 [95% CI, 0.86−1.14]). Results from the IVW, MR Egger, and PWM analyses are illustrated in Figure 2.

**Figure 3:**
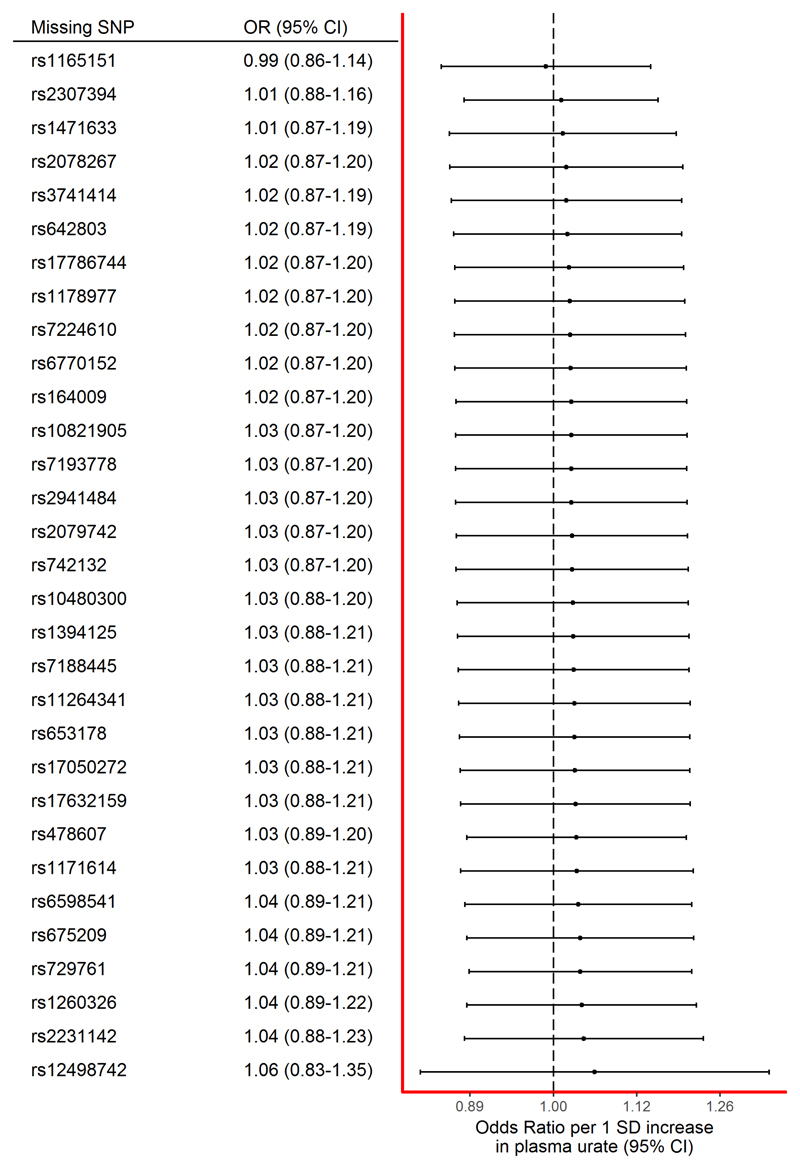
Forest plot of the results of the leave-one-out sensitivity analysis, where each SNP in the instrument was iteratively removed from the instrument. CI = confidence interval; OR = odds ratio; SD = standard deviation; SNP = single-nucleotide polymorphism. [Color figure can be viewed at www.annalsofneurology.org]

The effect estimate stayed consistent regardless of individual SNPs being removed from the instrument (Fig 3). Notably, even leaving the SNP with the narrowest confidence intervals (rs12498742) out of the instrument did not dramatically alter the effect estimate.

Our study had a power of 80% to detect a true causal effect of a relative difference of 10% per 1-SD change in urate (ie, OR <0.9 or .>1.1).

The study was repeated using recently published PD GWAS meta-analysis summary statistics by Chang et al and yielded a quantitatively and qualitatively similar result (IVW effect estimate OR, 0.99; 95% CI, 0.87–1.13).^35^

## Discussion

In this study, we investigated the causal relationship between urate and risk of PD, using two-sample MR. We compared results from three MR analytical approaches, each with different underlying assumptions, to explore the validity of our instruments and the effect estimates they produced. To our knowledge, this is the largest-scale MR study of this association to date. Our results found no causal link between plasma urate levels and risk of PD, which suggests that the associations between urate and PD risk reported previously could be attributed to confounding or reverse causality. However, factors that cause or protect against a disease may be different from those that affect prognosis once a disease is established.^36^,^37^ The results presented here are not evidence against urate being effective in slowing disease progression in people that already have a diagnosis of PD. Thus, current trials of inosine, a urate precursor, in patients with PD should not necessarily be influenced by our findings.^15^,^16^

In terms of observational data on the relationship between urate and PD, a meta-analysis of prevalent case-control studies which included 1,217 cases and 1,276 controls reported a standardized mean difference of −0.52 (95% CI, −0.72 to −0.31) in urate levels between PD cases and healthy controls, but these findings cannot distinguish between urate levels protecting against PD or PD causing a lowering of urate (reverse causality).^12^ A separate meta-analysis of six observational studies of incident cases, with 594 PD cases and 33,185 controls, reported a relative risk of PD of 0.65 (95% CI, 0.43–0.97) for “high” (≥6.8mg/dl) versus “low” serum urate, with some suggestion of a stronger effect in men compared to women.^13^ The results were essentially the same when analyses were restricted to those studies that had excluded cases occurring in the early years of follow-up, suggesting that the results were unlikely to be explained by reverse causality. However, there was significant heterogeneity between studies included in the meta-analyses (I^2^ = 75.6% and 43.1%, respectively). A nested prospective case-control study, which meta-analysed results from 388 new cases and 1,267 controls with three previously published prospective studies, focused on sex differences. This study reported an OR of 0.63 (95% CI, 0.42–0.95) in men and 0.89 (95% CI, 0.57–1.40) in women, when comparing those in the top quarter of urate levels to those in the bottom quarter.^14^ The primary studies included in these meta-analyses were all adjusted at least for age and smoking, but residual confounding could have been present.

A recent umbrella review of observational studies of environmental risk factors for PD judged the evidence for urate to be Class II. Despite consistent evidence for an association with PD, there was significant between-study heterogeneity, small-study effects, and the 95% prediction interval, a measure of the expected uncertainty in a future study on the same association, included the null value.^4^ In combination, these observations raise some doubts about the potential neuroprotective benefits of increasing circulating urate. Furthermore, in a large-scale hospital database study, no long-term protective effect was observed between gout and PD, whereas a diagnosis of PD was associated with a significant decrease in the subsequent risk of gout, suggesting that decreased urate levels are a feature of PD rather than a protective factor.^38^ Recently, changes in microbiota have been proposed as affecting both circulating urate levels and PD risk, and may possibly confound previous observational study associations.^39^

To our knowledge, there have been four previous studies examining the relationship between urate and PD using an MR-like approach; two have looked at the association with PD risk, one with PD progression and one with age at onset. Gao et al examined the association of 12 SNPs in the *SLC2A9* locus with PD in a case-control study in individuals of European descent, consisting of 788 self-reported cases and 911 controls.^20^ SNPs were all in linkage disequilibrium (R^2^ > 0.7), and the study found that for one SNP, the allele associated with lower plasma urate, was nominally associated with a higher risk of PD (95% CI, 1.48 (1.01–2.16); *p* = 0.04). This association did not survive multiple testing correction. González-Aramburu et al constructed an unweighted allele score using SNPs from eight independent loci in 1,061 Spanish cases and 754 controls and found that those with 10 to 15 urate-decreasing alleles (compared to those with seven or fewer) had increased odds of PD (95% CI, 1.55 [1.10–2.18]).^19^

Simon et al used MR to explore the effect of urate on PD progression; they used three SNPs in linkage disequilibrium in the *SLC2A9* locus to stratify PD patients into three groups based on the number of risk alleles, and used time to initiation of levodopa treatment as the outcome in 735 PD patients of European descent.^17^ They observed a protective effect of urate on PD progression with a hazard ratio of 1.27 (95% CI = 1.00–1.61; *p* = 0.0497) for a 0.5-mg/dl genetically conferred decrease in serum urate. In a further study of prognosis, Facheris et al looked at four SNPs in the *SLC2A9* locus, and their association with age at onset in 664 PD patients of European ancestry, and found one SNP to be associated with a mean difference in age at onset of −4.56 (95% CI, −8.13 to −1.00) per urate-decreasing allele.^18^ These studies, undertaken in patients that already have PD, may not be directly comparable with our research findings because, as already mentioned, risk factors for disease may differ from those that affect timing of disease diagnosis and its progression. Thus, whereas these studies are relatively small and need further replication, it is possible that urate protects against a more-rapid progression, but that it has no effect on whether one gets PD or not (as indicated by our study).

Using only the *SLC2A9* locus as the instrument, as some previous studies have done, makes it impossible to differentiate whether the MR effect estimate is for changes in urate, or some other mechanism in which the *SLC2A9* transporter is involved. With a multilocus instrument, although the individual loci in the instrument may act through different, possibly pleiotropic, mechanisms, their shared effect is through altered circulating urate concentration. Furthermore, in our study, when using only the SNP from the *SLC2A9* locus (rs12498742) as the instrumental variable for the association between urate and PD, the causal effect estimate is still a clear null (OR, 1.00 [95% CI, 0.86−1.17]).

### Strengths and Limitations

Key strengths of our study are its large sample size with 13,708 PD cases and the use of SNPs from 31 independent loci, which increases statistical power and allows the use of different methods for assessing potential bias attributed to pleiotropy. By using genetic instrumental variables that have been shown to be robustly associated with urate and replicate across studies, we are unlikely to have violated the first assumption of instrumental variable analysis. Although the use of aggregate data precludes us from examining whether the SNPs we have used associate with confounders of the urate-PD association, there is empirical evidence that in general genetic variants are less likely to be associated with common confounders than (nongenetic) risk factors.^21^,^38^ Last, the consistency of findings across three different MR methods, each with different assumptions regarding pleiotropy, suggests that bias was unlikely.

Limitations of our study include those related to the use of aggregate data, which means we cannot explore any potential nonlinear effects or whether there might be differences in effect between different groups (eg, between females and males, which has been suggested previously^14^). There is some evidence that the relationship between urate and PD may be more complex than previously reported, such as a U-shaped association.^39^ Finally, given that we have used a case-control study, our results might be influenced by survival bias if plasma urate concentrations affect mortality before patients are diagnosed with PD.

In conclusion, we do not find clear evidence for a linear causal protective effect of urate on PD risk. These findings should help in understanding PD pathogenesis and prioritizing potential disease-modifying treatments.

## Supplementary Material

Additional supporting information may be found online in the Supporting Information section at the end of the article.

Supplementary information

## Figures and Tables

**Table 1:** SNPs Used to Construct the Instrumental Variable

SNP	Nearest Gene	EA	OA	EAF	Beta	SE
rs1471633	*PDZK1*	A	C	0.46	0.037	0.0033
rs1260326	*GCKR*	T	C	0.41	0.045	0.0032
rs12498742	*SLC2A9*	A	G	0.77	0.236	0.0034
rs2231142	*ABCG2*	T	G	0.11	0.124	0.0051
rs675209	*RREB1*	T	C	0.27	0.036	0.0039
rs1165151	*SLC17A1*	T	G	0.47	–0.051	0.0028
rs1171614	*SLC16A9*	T	C	0.22	–0.052	0.0046
rs2078267	*SLC22A11*	T	C	0.51	–0.048	0.0038
rs478607	*NRXN2*	A	G	0.84	–0.017	0.0037
rs3741414	*INHBC*	T	C	0.24	–0.043	0.0045
rs11264341	*TRIM46*	T	C	0.43	–0.033	0.0039
rs17050272	*INHBB*	A	G	0.43	0.023	0.0039
rs6770152	*SFMBT1*	T	G	0.58	–0.029	0.0033
rs17632159	*TMEM171*	C	G	0.31	–0.026	0.0039
rs729761	*VEGFA*	T	G	0.3	–0.031	0.0039
rs1178977	*BAZ1B*	A	G	0.81	0.031	0.0046
rs10480300	*PRKAG2*	T	C	0.28	0.023	0.0039
rs2941484	*HNF4G*	T	C	0.44	0.029	0.0033
rs10821905	*A1CF*	A	G	0.18	0.037	0.0046
rs642803	*OVOL1*	T	C	0.46	–0.024	0.0033
rs653178	*ATXN2*	T	C	0.51	–0.023	0.0033
rs1394125	*UBE2Q2*	A	G	0.34	0.028	0.0039
rs6598541	*IGF1R*	A	G	0.36	0.028	0.0039
rs7193778	*NFAT5*	T	C	0.86	–0.030	0.0052
rs7188445	*MAF*	A	G	0.33	–0.021	0.0033
rs7224610	*HLF*	A	C	0.58	–0.028	0.0033
rs742132	*LRRC16A*	A	G	0.7	0.035	0.0060
rs2307394	*ORC4L*	T	C	0.68	–0.019	0.0033
rs17786744	*STC1*	A	G	0.58	–0.019	0.0033
rs2079742	*BCAS3*	T	C	0.85	0.028	0.0052
rs164009	*QRICH2*	A	G	0.61	0.018	0.0033

SNPs = single-nucleotide polymorphisms; EA = effect allele; OA = other allele; EAF = effect allele frequency; Beta = SD change in urate per effect allele; SE = standard error.
