# Supplementary material for: Mendelian Randomization Study Shows No Causal Relationship Between Circulating Urate Levels and Parkinson’s Disease
Source: Ann Neurol. Author manuscript; Available in PMC 2019 Apr 24. (PMC6481555; doi:10.1002/ana.25294)
Supplement: Supplementary information [file NIHMS82521-supplement-Supplementary_information.docx]

**Supplementary material:**

**Mendelian randomization study shows no causal relationship between circulating urate levels and Parkinson’s disease**

**Kia DA et al**

Contents:

Page 2: Supplementary table 1. IPDGC consortium members and affiliations

Page 7: Supplementary table 2. IPDGC acknowledgements

**Supplementary table 1. IPDGC consortium members and affiliations:**

| **United Kingdom** | |
| --- | --- |
| **Member** | **Institution** |
| Alastair J Noyce | Preventive Neurology Unit, Wolfson Institute of Preventive Medicine, QMUL, London, UK and Department of Molecular Neuroscience, UCL, London, UK |
| Arianna Tucci | Department of Molecular Neuroscience, UCL Institute of Neurology, London, UK |
| Demis A Kia | UCL Genetics Institute; and Department of Molecular Neuroscience, UCL Institute of Neurology, London, UK |
| Gavin Charlesworth | Department of Molecular Neuroscience, UCL Institute of Neurology, London, UK |
| Manuela Tan | Department of Clinical Neuroscience, University College London, London, UK |
| Henry Houlden | Department of Molecular Neuroscience, UCL Institute of Neurology, London, UK |
| Huw R Morris | Department of Clinical Neuroscience, University College London, London, UK |
| Helene Plun-Favreau | Department of Molecular Neuroscience, UCL Institute of Neurology, London, UK |
| Peter Holmans | Biostatistics & Bioinformatics Unit, Institute of Psychological Medicine and Clinical Neuroscience, MRC Centre for Neuropsychiatric Genetics & Genomics, Cardiff, UK |
| John Hardy | Department of Molecular Neuroscience, UCL Institute of Neurology, London, UK |
| Jose Bras | UK Dementia Research Institute at UCL and Department of Molecular Neuroscience, UCL Institute of Neurology, London, UK |
| John Quinn | Institute of Translational Medicine, University of Liverpool, Liverpool, UK |
| Kin Y Mok | Department of Molecular Neuroscience, UCL Institute of Neurology, London, UK |
| Kerri J. Kinghorn | Institute of Healthy Ageing, University College London, London, UK |
| Kimberley Billingsley | Institute of Translational Medicine, University of Liverpool, Liverpool, UK |
| Nicholas W Wood | UCL Genetics Institute; and Department of Molecular Neuroscience, UCL Institute of Neurology, London, UK |
| Patrick Lewis | University of Reading, Reading, UK |
| Rita Guerreiro | UK Dementia Research Institute at UCL and Department of Molecular Neuroscience, UCL Institute of Neurology, London, UK |
| Ruth Lovering | University College London, London, UK |
| Raquel Duran Ogalla | University College London, London, UK |
| Lea R’Bibo | Department of Molecular Neuroscience, UCL Institute of Neurology, London, UK |
| Mie Rizig | Department of Molecular Neuroscience, UCL Institute of Neurology, London, UK |
| Mina Ryten | Department of Molecular Neuroscience, UCL Institute of Neurology, London, UK |
| Valentina Escott-Price | MRC Centre for Neuropsychiatric Genetics and Genomics, Cardiff University School of Medicine, Cardiff, UK |
| Viorica Chelban | Department of Molecular Neuroscience, UCL Institute of Neurology, London, UK |
| Thomas Foltynie | UCL Institute of Neurology, London, UK |
| Una-Marie Sheerin | Department of Molecular Neuroscience, UCL Institute of Neurology, London, UK |
| Nigel Williams | MRC Centre for Neuropsychiatric Genetics and Genomics, Cardiff, UK |
|  | |
| **France** | |
| **Member** | **Institution** |
| Alexis Brice | Institut du Cerveau et de la Moelle épinière, ICM, Inserm U 1127, CNRS, UMR 7225, Sorbonne Universités, UPMC University Paris 06, UMR S 1127, AP-HP, Pitié-Salpêtrière Hospital, Paris, France |
| Fabrice Danjou | Institut du Cerveau et de la Moelle épinière, ICM, Inserm U 1127, CNRS, UMR 7225, Sorbonne Universités, UPMC University Paris 06, UMR S 1127, AP-HP, Pitié-Salpêtrière Hospital, Paris, France |
| Suzanne Lesage | Institut du Cerveau et de la Moelle épinière, ICM, Inserm U 1127, CNRS, UMR 7225, Sorbonne Universités, UPMC University Paris 06, UMR S 1127, AP-HP, Pitié-Salpêtrière Hospital, Paris, France |
| Jean-Christophe Corvol | Institut du Cerveau et de la Moelle épinière, ICM, Inserm U 1127, CNRS, UMR 7225, Sorbonne Universités, UPMC University Paris 06, UMR S 1127, Centre d’Investigation Clinique Pitié Neurosciences CIC-1422, AP-HP, Pitié-Salpêtrière Hospital, Paris, France |
| Maria Martinez | INSERM UMR 1220; and Paul Sabatier University, Toulouse, France |
|  | |
| **Germany** | |
| **Member** | **Institution** |
| Anamika Giri | Department for Neurodegenerative Diseases, Hertie Institute for Clinical Brain Research, University of Tübingen, and DZNE, German Center for Neurodegenerative Diseases, Tübingen, Germany |
| Claudia Schulte | Department for Neurodegenerative Diseases, Hertie Institute for Clinical Brain Research, University of Tübingen, and DZNE, German Center for Neurodegenerative Diseases, Tübingen, Germany |
| Kathrin Brockmann | Department for Neurodegenerative Diseases, Hertie Institute for Clinical Brain Research, University of Tübingen, and DZNE, German Center for Neurodegenerative Diseases, Tübingen, Germany |
| Javier Simón-Sánchez | Department for Neurodegenerative Diseases, Hertie Institute for Clinical Brain Research, University of Tübingen, and DZNE, German Center for Neurodegenerative Diseases, Tübingen, Germany |
| Peter Heutink | DZNE, German Center for Neurodegenerative Diseases and Department for Neurodegenerative Diseases, Hertie Institute for Clinical Brain Research, University of Tübingen, Tübingen, Germany |
| Patrizia Rizzu | DZNE, German Center for Neurodegenerative Diseases |
| Manu Sharma | Centre for Genetic Epidemiology, Institute for Clinical Epidemiology and Applied Biometry, University of Tubingen, Germany |
| Thomas Gasser | Department for Neurodegenerative Diseases, Hertie Institute for Clinical Brain Research, and DZNE, German Center for Neurodegenerative Diseases, Tübingen, Germany |
|  | |
| **United States of America** | |
| **Member** | **Institution** |
| Aude Nicolas | Laboratory of Neurogenetics, National Institute on Aging, Bethesda, MD, USA |
| Mark R Cookson | Laboratory of Neurogenetics, National Institute on Aging, Bethesda, USA |
| Sara Bandres-Ciga | Laboratory of Neurogenetics, National Institute on Aging, Bethesda, MD, USA |
| Cornelis Blauwendraat | National Institute on Aging and National Institute of Neurological Disorders and Stroke, USA |
| Faraz Faghri | Laboratory of Neurogenetics, National Institute on Aging, Bethesda, USA; Department of Computer Science, University of Illinois at Urbana-Champaign, Urbana, IL, USA |
| J Raphael Gibbs | Laboratory of Neurogenetics, National Institute on Aging, National Institutes of Health, Bethesda, MD, USA |
| Dena G Hernandez | Laboratory of Neurogenetics, National Institute on Aging, Bethesda, MD, USA |
| Joshua M. Shulman | Baylor College of Medicine, Houston, Texas, USA |
| Mike A. Nalls | Laboratory of Neurogenetics, National Institute on Aging, Bethesda, USA; CEO/Consultant Data Tecnica International, Glen Echo, MD, USA |
| Laurie Robak | Baylor College of Medicine, Houston, Texas, USA |
| Steven Lubbe | Ken and Ruth Davee Department of Neurology, Northwestern University Feinberg School of Medicine, Chicago, IL, USA |
| Steven Finkbeiner | Departments of Neurology and Physiology, University of California, San Francisco; Gladstone Institute of Neurological Disease; Taube/Koret Center for Neurodegenerative Disease Research, San Francisco, CA, USA |
| Niccolo E. Mencacci | Northwestern University Feinberg School of Medicine, Chicago, IL, USA |
| Codrin Lungu | National Institutes of Health Division of Clinical Research, NINDS, National Institutes of Health, Bethesda, MD, USA |
| Andrew B Singleton | Laboratory of Neurogenetics, National Institute on Aging, Bethesda, MD, USA |
| Sonja Scholz | Neurodegenerative Diseases Research Unit, National Institute of Neurological Disorders and Stroke, Bethesda, MD, USA |
| Xylena Reed | Laboratory of Neurogenetics, National Institute on Aging, Bethesda, MD, USA |
|  | |
| **Canada** | |
| **Member** | **Institution** |
| Ziv Gan-Or | Montreal Neurological Institute and Hospital, Department of Neurology & Neurosurgery, Department of Human Genetics, McGill University, Montréal, QC, H3A 0G4, Canada |
| Guy A. Rouleau | Montreal Neurological Institute and Hospital, Department of Neurology & Neurosurgery, Department of Human Genetics, McGill University, Montréal, QC, H3A 0G4, Canada |
|  |  |
| The Netherlands |  |
| Member | Institution |
| Jacobus J van Hilten | Department of Neurology, Leiden University Medical Center, Leiden, Netherlands |
| Johan Marinus | Department of Neurology, Leiden University Medical Center, Leiden, Netherlands |
|  | |
| **Spain** | |
| **Member** | **Institution** |
| Juan A. Botía | Universidad de Murcia, Murcia, Spain |
| Jordi Clarimón | Memory Unit, Department of Neurology, IIB Sant Pau, Hospital de la Santa Creu i Sant Pau, Universitat Autònoma de Barcelona, Barcelona, and Centro de Investigación Biomédica en Red en Enfermedades Neurodegenerativas (CIBERNED), Madrid |
| Oriol Dols-Icardo | Memory Unit, Department of Neurology, IIB Sant Pau, Hospital de la Santa Creu i Sant Pau, Universitat Autònoma de Barcelona, Barcelona, and Centro de Investigación Biomédica en Red en Enfermedades Neurodegenerativas (CIBERNED), Madrid |
| Jaime Kulisevsky | Movement Disorders Unit, Department of Neurology, IIB Sant Pau, Hospital de la Santa Creu i Sant Pau, Universitat Autònoma de Barcelona, Barcelona, and Centro de Investigación Biomédica en Red en Enfermedades Neurodegenerativas (CIBERNED), Madrid |
| Javier Pagonabarraga | Movement Disorders Unit, Department of Neurology, IIB Sant Pau, Hospital de la Santa Creu i Sant Pau, Universitat Autònoma de Barcelona, Barcelona, and Centro de Investigación Biomédica en Red en Enfermedades Neurodegenerativas (CIBERNED), Madrid |
| Juan Marín | Movement Disorders Unit, Department of Neurology, IIB Sant Pau, Hospital de la Santa Creu i Sant Pau, Universitat Autònoma de Barcelona, Barcelona, and Centro de Investigación Biomédica en Red en Enfermedades Neurodegenerativas (CIBERNED), Madrid |
|  | |
| **Austria** | |
| **Member** | **Institution** |
| Alexander Zimprich | Department of Neurology, Medical University of Vienna, Austria) |
|  | |
| **Norway** | |
| **Member** | **Institution** |
| Lasse Pihlstrom | Department of Neurology, Oslo University Hospital, Oslo, Norway |
|  | |
| **Estonia** | |
| **Member** | **Institution** |
| Sulev Koks | Department of Pathophysiology, University of Tartu, Tartu, Estonia |
| Pille Taba | Department of Neurology and Neurosurgery, University of Tartu, Tartu, Estonia |

**Supplementary table 2. IPDGC acknowledgements:**

| This work was supported in part by the Intramural Research Programs of the National Institute of Neurological Disorders and Stroke (NINDS), the National Institute on Aging (NIA), and the National Institute of Environmental Health Sciences both part of the National Institutes of Health, Department of Health and Human Services; project numbers 1ZIA-NS003154, Z01-AG000949-02 and Z01-ES101986. |
| --- |
| This work was supported by the Department of Defense (award W81XWH-09-2-0128), and The Michael J Fox Foundation for Parkinson’s Research. |
| This work was supported by National Institutes of Health grants R01NS037167, R01CA141668, P50NS071674, American Parkinson Disease Association (APDA); Barnes Jewish Hospital Foundation; Greater St Louis Chapter of the APDA; Hersenstichting Nederland; the Prinses Beatrix Fonds. |
| The KORA (Cooperative Research in the Region of Augsburg) research platform was started and financed by the Forschungszentrum für Umwelt und Gesundheit, which is funded by the German Federal Ministry of Education, Science, Research, and Technology and by the State of Bavaria. |
| This study was also funded by the German Federal Ministry of Education and Research (BMBF) under the funding code 031A430A, the EU Joint Programme - Neurodegenerative Diseases Research (JPND) project under the aegis of JPND -www.jpnd.eu- through Germany, BMBF, funding code 01ED1406 and iMed - the Helmholtz Initiative on Personalized Medicine. |
| This study is funded by the German National Foundation grant (DFG SH599/6-1) (grant to M.S), Michael J Fox Foundation, and MSA Coalition, USA (to M.S). |
| The French GWAS work was supported by the French National Agency of Research (ANR-08-MNP-012). |
| This study was also funded by France-Parkinson Association, Fondation de France, the French program “Investissements d’avenir” funding (ANR-10-IAIHU-06) and a grant from Assistance Publique-Hôpitaux de Paris (PHRC, AOR-08010) for the French clinical data. |
| This study was also sponsored by the Landspitali University Hospital Research Fund (grant to SSv); Icelandic Research Council (grant to SSv); and European Community Framework Programme 7, People Programme, and IAPP on novel genetic and phenotypic markers of Parkinson’s disease and Essential Tremor (MarkMD), contract number PIAP-GA-2008-230596 MarkMD (to HP and JHu). |
| Institutional research funding IUT20-46 was received of the Estonian Ministry of Education and Research (SK). |
| The McGill study was funded by the Michael J. Fox Foundation and the Canadian Consortium on Neurodegeneration in Aging (CCNA). |
| This study utilized the high-performance computational capabilities of the Biowulf Linux cluster at the National Institutes of Health, Bethesda, Md. (http://biowulf.nih.gov), and DNA panels, samples, and clinical data from the National Institute of Neurological Disorders and Stroke Human Genetics Resource Center DNA and Cell Line Repository. |
| People who contributed samples are acknowledged in descriptions of every panel on the repository website. |
| We thank the French Parkinson’s Disease Genetics Study Group and the Drug Interaction with genes (DIGPD) study group: Y Agid, M Anheim, F Artaud, A-M Bonnet, C Bonnet, F Bourdain, J-P Brandel, C Brefel-Courbon, M Borg, A Brice, E Broussolle, F Cormier-Dequaire, J-C Corvol, P Damier, B Debilly, B Degos, P Derkinderen, A Destée, A Dürr, F Durif, A Elbaz, D Grabli, A Hartmann, S Klebe, P. Krack, J Kraemmer, S Leder, S Lesage, R Levy, E Lohmann, L Lacomblez, G Mangone, L-L Mariani, A-R Marques, M Martinez, V Mesnage, J Muellner, F Ory-Magne, F Pico, V Planté-Bordeneuve, P Pollak, O Rascol, K Tahiri, F Tison, C Tranchant, E Roze, M Tir, M Vérin, F Viallet, M Vidailhet, A You. |
| We also thank the members of the French 3C Consortium: A Alpérovitch, C Berr, C Tzourio, and P Amouyel for allowing us to use part of the 3C cohort, and D Zelenika for support in generating the genome-wide molecular data. |
| We thank P Tienari (Molecular Neurology Programme, Biomedicum, University of Helsinki), T Peuralinna (Department of Neurology, Helsinki University Central Hospital), L Myllykangas (Folkhalsan Institute of Genetics and Department of Pathology, University of Helsinki), and R Sulkava (Department of Public Health and General Practice Division of Geriatrics, University of Eastern Finland) for the Finnish controls (Vantaa85+ GWAS data). |
| We used genome-wide association data generated by the Wellcome Trust Case-Control Consortium 2 (WTCCC2) from UK patients with Parkinson’s disease and UK control individuals from the 1958 Birth Cohort and National Blood Service. |
| Genotyping of UK replication cases on ImmunoChip was part of the WTCCC2 project, which was funded by the Wellcome Trust (083948/Z/07/Z). |
| UK population control data was made available through WTCCC1. |
| This study was supported by the Medical Research Council and Wellcome Trust disease centre (grant WT089698/Z/09/Z to NW, JHa, and ASc). |
| As with previous IPDGC efforts, this study makes use of data generated by the Wellcome Trust Case-Control Consortium. |
| A full list of the investigators who contributed to the generation of the data is available from www.wtccc.org.uk. |
| Funding for the project was provided by the Wellcome Trust under award 076113, 085475 and 090355. |
| This study was also supported by Parkinson’s UK (grants 8047 and J-0804) and the Medical Research Council (G0700943 and G1100643). |
| Sequencing and genotyping done in McGill University was supported by grants from the Michael J Fox Foundation and the Canadian Consortium on Neurodegeneration in Aging (CCNA). |
| We thank Jeffrey Barrett and Jason Downing for assistance with the design of the ImmunoChip and NeuroX arrays. |
| DNA extraction work that was done in the UK was undertaken at University College London Hospitals, University College London, who received a proportion of funding from the Department of Health’s National Institute for Health Research Biomedical Research Centres funding. |
| This study was supported in part by the Wellcome Trust/Medical Research Council Joint Call in Neurodegeneration award (WT089698) to the Parkinson’s Disease Consortium (UKPDC), whose members are from the UCL Institute of Neurology, University of Sheffield, and the Medical Research Council Protein Phosphorylation Unit at the University of Dundee. |
| We thank the Quebec Parkinson’s Network (http://rpq-qpn.org) and its members. |
| MN’s participation is supported by a consulting contract between Data Tecnica International and the National Institute on Aging, NIH, Bethesda, MD, USA |
